# Noma, a neglected disease: A viewpoint article

**DOI:** 10.1371/journal.pntd.0009437

**Published:** 2021-06-17

**Authors:** Elise Farley, Cono Ariti, Mohana Amirtharajah, Charity Kamu, Bukola Oluyide, Muhammad Shoaib, Shafiu Isah, Adeniyi Semiyu Adetunji, Fatima Saleh, Chikwe Ihekweazu, Monique Pereboom, Mark Sherlock

**Affiliations:** 1 Médecins Sans Frontières, Sokoto, Nigeria; 2 Noma Children’s Hospital, Sokoto, Nigeria; 3 Centre for Trials Research, College of Biomedical and Life Sciences, Cardiff University, Cardiff, United Kingdom; 4 Médecins Sans Frontières, Amsterdam, the Netherlands; 5 Nigeria Centre for Disease Control, Abuja, Nigeria; Yale University School of Medicine, UNITED STATES

## Introduction

Noma, also known as cancrum oris, is a gangrenous infection that causes rapid, widespread orofacial destruction [1]. The reported mortality rate for untreated patients is 90% within weeks after the onset of first symptoms [1]. Treatment in the early acute stages with antibiotics, wound debridement, and nutritional support greatly reduces mortality and morbidity [1]. The cause of noma is unknown, and noma is not contagious [2]. Those who survive the acute stages of noma face a lifetime of functional challenges such as difficulty eating and speaking [1]. Survivors and their families frequently suffer stigmatization [3]. Extensive reconstructive surgery is often utilized in an attempt to improve function and aesthetics for patients with sequelae [4]. Noma most frequently affects children between 2 and 5 years of age who live in low-income settings in Africa and Asia [1]. The World Health Organization (WHO) estimates that 140,000 new cases of noma occur annually [1]. The main risk factors for noma include chronic malnutrition, a lack of access to healthcare (specifically immunizations), and comorbidities such as measles and human immunodeficiency virus (HIV) [1]. WHO classifies noma into stages: Stage 0: simple gingivitis, Stage 1: acute necrotizing gingivitis, Stage 2: oedema, Stage 3: gangrene, Stage 4: scarring, and Stage 5: sequelae [1]. The disease was once frequently reported in Europe and North America, although as living conditions improved in these settings, noma has been eradicated (besides a few sporadic cases in immunocompromised individuals) [2].

Noma cases are frequently reported in Nigeria [2]. For the past 2 decades, the Nigerian Ministry of Health has run a specialized program at the Noma Children’s Hospital in Sokoto State in the northwest of the country, supported by Médecins Sans Frontières (MSF) since 2014. Activities include providing care for patients with acute noma (antibiotics, oral hygiene, treatment for underlying morbidities, wound debridement, and wound dressing), continuous care for patients with noma sequelae (surgical interventions and postoperative care), integrated hospital-based services (nutrition, mental health, physiotherapy, lab services, water and sanitation, and vaccinations), and community-based services (follow-up, active case finding, awareness raising, health promotion, and education).

This viewpoint article offers a summary of what is known about noma and suggestions for why noma is still neglected, despite the severe ramifications of the infection and the centuries of clinical reporting of the disease in the scientific literature. We explore the disability-adjusted life years (DALYs) for noma in northwest Nigeria and how these compare to diseases listed on WHO’s list of neglected tropical diseases (NTDs). We demonstrate the importance of recognizing noma as a neglected disease and offer ideas for the integration of noma into existing health structures. These suggestions could lead to the effective control of noma in a globally integrated manner.

### Neglected noma

Despite the major developments in health research since noma was first reported (by Hippocrates (460 to 370 BC) [5]), the disease remains poorly understood in the global health community [5]. The majority of research on noma are case reports, and there are several major gaps in knowledge, including a lack of understanding of the burden, distribution, community-level mortality rate, and aetiology of the disease [5]. Noma is a difficult disease to study due to its rapid progression, reported high mortality rate, and the remote location of affected patients [1]. Noma is infrequently taught in medical, nursing, or tropical medicine courses, and this leads to a lack of knowledge about noma among healthcare workers [6,7]. Affected patients often lack access to quality healthcare, and, even when healthcare is sought, noma is regularly misdiagnosed due to a lack of knowledge about noma among healthcare workers, leading to underreporting of the disease [6,7]. Patients with noma thus remain invisible within their communities, the health systems that are supposed to serve them, and the global community, feeding into the poor understanding of this disease and its subsequent neglect.

### DALYs

Noma often leads to death, and those who survive are frequently affected for the rest of their lives. To gain a better understanding of the impact of noma, we calculated the DALYs (years of life lost (number of deaths × standard life expectancy at age of death in years) + years lost due to disability (number of prevalent cases × disability weight)) for noma using the noma prevalence estimates attained from an MSF-supported community-based prevalence study in Sokoto and Kebbi States, northwest Nigeria (3,300 per 100,000 or 129,120 cases [8]) and the disability weight of cleft (disfigurement level 2 from the 2019 Global Burden of Disease Study (0.115) [9]). It was assumed death would occur between 1 and 4 years of age, and WHO life tables for Nigeria were used to ascertain the standard life expectancy at age of death in years (66.59 years) [10]. Two mortality rates were used in the calculations, 90% (untreated) [1] and 20% (treated) [11].

The DALY estimates show that noma patients in Sokoto and Kebbi states who do not receive treatment have much higher DALYs (198,152 DALYs/100,000; individual DALY 60) in comparison to those who do receive timely treatment (44,329 DALYs/100,000; individual DALY 13) (Table 1).

**Table 1:** Noma DALY estimates in millions per 100,000 children aged between 0 and 15 at the time of the survey (2018 [8]) and per individual in Sokoto and Kebbi states, northwest Nigeria.

	DALYs in millions (95% CI)	DALY per 100,000	Individual DALY
Untreated mortality 90%	7.8 (CI 6.3, 9.2)	198,152	60
Treated mortality 20%	1.7 (CI 1.4, 2.0)	44,329	13

DALY, disability-adjusted life year.

The estimates are comparable and frequently greater than diseases recognized by WHO as neglected (Table 2).

**Table 2:** DALYs from the 2019 Global Burden of Disease Study for WHO-recognized NTDs [12].

Disease	DALYs in millions (95% CI)
Scabies and other ectoparasitoses	4.8 (CI 2.7, 7.7)
Dengue	2.4 (CI 0.9, 3.3)
Lymphatic filariasis	1.6 (CI 0.9, 2.7)
Schistosomiasis	1.6 (CI 1.0, 2.6)
Taeniasis/cysticercosis	1.4 (CI 0.9, 1.9)
Onchocerciasis	1.2 (CI 0.8, 1.8)
Rabies	0.8 (CI 0.3, 1.1)
Leishmaniasis	0.7 (CI 0.4, 1,6)
Chagas disease	0.3 (CI 0.2, 0.5)
Trachoma	0.2 (CI 0.1, 0.3)
African trypanosomiasis	0.1 (CI 0.04, 0.2)
Echinococcosis	0.1 (CI 0.09, 0.2)
Leprosy	0.02 (CI 0.01, 0.04)
Buruli ulcer	Not available
Dracunculiasis	Not available
Foodborne trematodiases	Not available
Mycetoma, chromoblastomycosis, and other deep mycoses	Not available
Snakebite envenoming	Not available
Soil-transmitted helminthiases	Not available
Yaws	Not available

DALY, disability-adjusted life year; NTD, neglected tropical disease; WHO, World Health Organization.

There are several limitations to DALY calculations for NTDs in general [13] and specifically for the above noma calculations: Using cleft as a proxy could underestimate the impact of noma, and the mortality rates used are not based on robust evidence and could alter our findings. Despite these limitations, the DALY estimates show that noma has a long-lasting impact on individuals affected by the disease, and therefore noma should receive increased attention—through efforts to prevent and treat this disease.

### Action points

There are several actions that can be taken to prevent new cases from developing, increase awareness about noma, and improve case detection and reporting.

1. Due to the dire ramifications of noma, the first action point, and the ideal approach to control the disease, is through prevention—and removing the main risk factors for infection. Prevention initiatives could include increasing uptake of childhood vaccinations, feeding programs in areas with high levels of malnutrition, and improving access to oral healthcare. These measures would reduce the incidence of noma and multiple other childhood diseases.
2. The second is the addition of noma to WHO’s list of NTDs. This addition will highlight the neglected nature of noma and raise awareness about the disease on the global stage. Furthermore, this recognition will provide the impetus to increase funding for prevention and treatment programs and increase the amount of research that is conducted on noma. This additional research is needed in order to fill the gaps in knowledge about the disease, which will be beneficial for prevention, early detection, policy planning, resource allocation, and program planning.
3. A further action is for governments and nongovernmental organizations to improve surveillance initiatives by integrating noma case detection into existing health activities. This could be achieved by including oral screenings in skin NTD activities, routine primary healthcare assessments, malnutrition surveys, vaccination campaigns, and mass drug administration programs for other NTDs.
4. An additional action is to include noma in medical, nursing, community health officer, and community health extension worker training programs. This will improve the healthcare received by patients and the efficiency of referrals. Improved access to appropriate treatments in the early stages of noma will likely lead to improved morbidity and mortality.

## Conclusions

Noma is a rapidly progressing infection that affects vulnerable children living in areas with limited access to quality healthcare. The infection has dire, often catastrophic consequences for the patient and their families. These impacts, when quantified using DALYS, surpass many diseases already recognized by WHO as NTDs. The neglect of noma by the global community should be rectified. There are several initiatives that can be taken to integrate noma prevention and surveillance activities into existing health programs. This integrated approach should lead to a reduced prevalence of noma and improved outcomes.
